# Rapidly progressive glomerulonephritis in a child with Henoch-Schönlein Vasculitis and familial Mediterranean fever

**DOI:** 10.1186/1546-0096-7-8

**Published:** 2009-05-07

**Authors:** Betul Sozeri, Sevgi Mir, Pelin Ertan, Orhan Deniz Kara, Sait Sen

**Affiliations:** 1 Department of Pediatric Nephrology, Faculty of Medicine, Ege University, Izmir, Turkey; 2 Department of Pediatric Nephrology, Faculty of Medicine, Celal Bayar University, Manisa, Turkey; 3 Department of Pathology, Faculty of Medicine, Ege University, Izmir, Turkey

## Abstract

Henoch-Schonlein Vasculitis (HSV) is systemic small vessel vasculitis involving the skin, kidney, joints, and gastrointestinal tract. The proportion of patients reported to have renal involvement varies between 20% and 80%. Rapidly progressive glomerulonephritis (RPGN)is rare syndrome in children, characterized by clinical features of glomerulonephritis (GN) and rapid loss of renal function. We present a severe kidney involvement in a 14 year old boy with HSV in who is carring MEFV mutation. A 14 year old boy had developed sudden onset of palpable purpuric rash on his extensor surfaces of lower extremities. He had elevated an erythrocyte sedimentation rate (ESR) (45 mm/h), C-reactive protein (3.74 mg/dl), serum urea 66 mg/dl, serum creatinine 1.8 mg/dl. Also, he had hypocomplementemia. Antinuclear antibody, anti ds DNA, antineutrophil cytoplasmic antibody, anticardiolipine antibodies were negative. Urinalysis revealed macroscopic hematuria and proteinuria with a 24-h urinary protein excretion of 55 mg/m2/h. The renal biopsy specimen showed crescentic and necrotizing glomerulonephritis. He had also M694V/E148Q compound heterozygote mutation. Clinical symptoms and renal failure resolved with intermittant hemodialysis and medical therapy.

## Background

Henoch-Schönlein Vasculitis (HSV) is systemic small vessel vasculitis involving the skin, kidney, joints, and gastrointestinal tract. The annual incidence of HSV is 22 per 100000. The pathogenesis of HSV remains unknown; however, HSV is generally believed to be immune complex-mediated disease characterized by the presence of polymeric IgA1-containing immune complexes predominantly in dermal, gastrointestinal and glomeruler capillaries [1,2].

The proportion of patients reported to have renal involvement varies between 20% and 80% [3-5]. In 80% of children with a urinary abnormality, the first abnormality is detected within 4 weeks of onset of the illness [6]. Hematuria may occasionally be the initial feature. Common urinary abnormalities are albuminuria and microscopic hematuria. A smaller number of patients have macroscopic hematuria. Acute nephritic syndrome occurs in more severe cases and may lead to nephrotic syndrome or to renal insufficiency [6,7].

Rapidly progressive glomerulonephritis (RPGN) is rare syndrome in children, characterized by clinical features of glomerulonephritis (GN) and rapid loss of renal function [8]. This clinical course may be seen in any form of GN including poststreptococcal glomerulonephritis, renal vasculitis, HSV.

It has been reported that certain vasculitides such as HSV and polyarteritis nodosa (PAN) are more frequent among familial Mediterranean fever (FMF) patients [9-14].

We present a severe kidney involvement in a 14 year old boy with HSV in who carries MEFV mutation.

## Case presentation

A 14 year old boy was referred to hospital with 2 days history of back and calf pain. He had developed sudden onset of palpable purpuric rash on his extensor surfaces of lower extremities especially bilateral ankles on admission. There was no history of recent drug exposure, immunization, or upper respiratory tract infection. He denied recurrent attacks of abdominal pain and fever. Family history for FMF was negative. Physical examination showed a temperature of 38.3°C, respiratory rate of 48/min, pulse rate 128/min. He was hypertensive at 140/100 mmHg. In auscultation, lungs were clear and the heart sounds were normal. Abdominal palpation was normal. There were symmetric palpable purpuric rash on his lower extremities.

Laboratory tests showed an erythrocyte sedimentation rate (ESR) of 45 mm/h (normal: <20 mm/h), C-reactive protein of 3.74 mg/dl (normal: < 0.3 mg/dl), hemoglobin 10.7 g/dl, hematocrit 32.3%, white blood cell count (WBC) 11900/mm3 with normal differential count, platelet count 340000/mm3, serum urea 66 mg/dl, serum creatinine 1.8 mg/dl, albumine 3.5 g/dl, total cholesterol 106 mg/dl, triglycerides 57 mg/dl, calcium 8.7 mg/dl, sodium 140 mEq/L, potassium 4.7 mEq/L, chloride 107 mEq/L, alanine aminotransferase 23 U/l, aspartate aminotransferase 16 U/l. Serum complement-3 (C3) and complement-4 (C4) were decreased (85.4 mg/dl, 6.6 mg/dl, respectively). Serum immunglobulin (Ig) levels were normal. The anti-streptolysin – O titer was 100 Todd unit and throat culture was negative for group A *B*-hemolytic streptococcus. Antinuclear antibody, anti ds DNA, antineutrophil cytoplasmic antibody, anticardiolipine antibodies were negative. Urinalysis revealed macroscopic hematuria and proteinuria with a 24-h urinary protein excretion of 55 mg/m2/h. The fecal occult blood testing was positive. Renal ultrasonography (US) showed increased echogenicity (grade 2) in bilateral kidneys. A skin biopsy showed a leucytoclastic vasculitis and deposition of IgA. Renal doppler US was normal. Renal magnetic resonance angiography (MRA) to rule out PAN revealed normal results. Based on these clinical findings, the patient was diagnosed as having HSV with renal, skin and probable gastrointestinal tract involvement. A diagnosis of FMF was suspected because of the absence of occult blood in the stool and normal mesenteric MRA. Since there are reports of increase frequency of MEFV mutation in patients with HSV, we performed DNA analysis in our patient and found that he was compound heterozygote, carrying M694V/E148Q.

**Figure 1:**
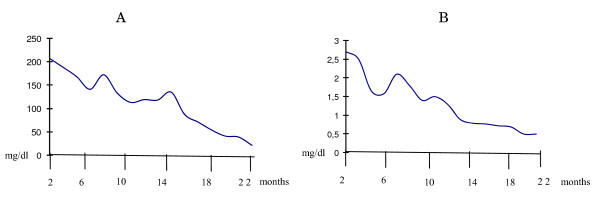
**Serum urea levels (A) and creatinine levels (B)**.

On the second day of hospitalization, He had periorbital and pitting pretibial edema. Respiratory examination revealed bibasilar crackles. His urine output decreased from 1.1 to 0.6 cc/kg/h and serum urea and creatine levels were increased (105 mg/dl, 3.5 mg/dl, respectively). We showed that serum urea and creatinine levels in figure 1. Echocardiography showed pericardial effusion (3 mm), minimal mitral regurgitation and increased inferior vena cava index and inferior vena cava collapsibility index.

**Figure 2:**
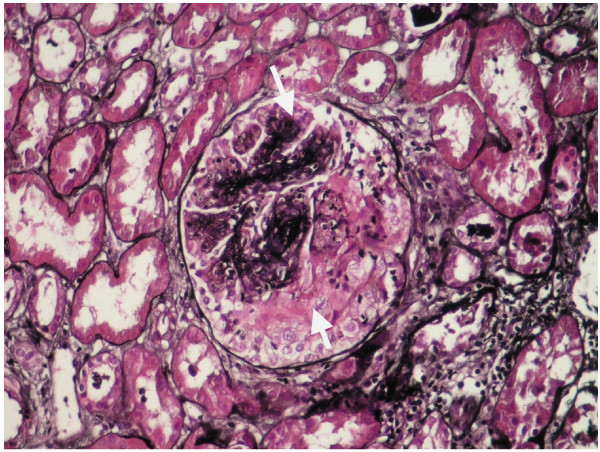
**Segmental, expansion of the mesangial matrix and epithelial cells prolipheration and/or necrotizing lesions**.

The renal biopsy specimen consists of two portions of renal cortex and medulla. Forty glomeruli and many arteries are available for examination on multiple sections. The glomeruli are not significantly enlarged. They show variable, mostly segmental, expansion of the mesangial matrix associated with mild segmental mesangial hypercellularity. 25–30% of 40 glomeruli show crescents and/or necrotizing lesions (figure 2). Another glomeruli show neutrophilic infiltration five glomeruli are available for examination by immunofluorescence. The mesangium contains peripheral membranous granular deposits of IgG (2+), IgA (3+), C3 (3+).

On the fifth day at hospitalization, hemodialysis was initiated for treatment of RPGN. Intravenous pulse methlyprednisolone was given (30 mg/kg, six consecutive days), followed by oral prednisolone and cyclophosphamide (2 mg/kg/day) for crescentic and necrotizing glomerulonephritis, in addition to Colchicine for FMF.

Clinical symptoms and renal failure resolved with intermittent hemodialysis therapy (total 20 cycles, twice a week). On one and half month after hospitalization, his abnormal serum and urinary findings resolved.

## Discussion

Henoch-Schönlein Vasculitis (HSV) is the most common vasculitis in childhood and is characterized by a systemic leukocytoclastic angiitis, mainly affecting the small vessels of the skin, joints, gastrointestinal tract, and kidneys. Other organs, such as brain, lungs, and scrotum, may occasionally be involved. HSV usually affects children between the ages of 5 and 15 years, while it is rare in adults and in infants [15,16].

Renal involvement has been reported to occur in 20–80% of children with HSV; among these Henoch-Schönlein nephritis patients, 1–7% would suffer from ESRD [4,5,17]. It has been generally accepted that the long-term outcome is determined by the severity of renal involvement, diffuse crescentic nephritis being associated with the worst outcome [18]. A small percentage of children develop serious renal disease. Essentially, renal involvement accounts for the major morbidity of the disease. RPGN is clinical syndrome characterized by an acute nephritic illness accompanied by a rapid loss of renal function over days to weeks [19]. The histopathological correlate is the presence of cresents involving 50% or more glomeruli. RPGN may ocur in a number of conditions including postinfectious GN and HSV [8,19,20].

We report a case of HSV complicated by RPGN. The presence of typical rash, severe nephritis and IgA deposits on skin and kidney biopsy confirm the diagnosis of HSV. In addition to the highly suggestive biopsy findings for HSV, negative ANCA, normal renal doppler USG, normal Renal MR angiography excluded the diagnosis of PAN.

The overall incidence of vasculitis in FMF patients is 1% of PAN and 5% of HSP, and it is significantly higher in FMF patients than in normal population [9-11,21,22].

The pathogenesis of vasculitis in patients with FMF is unknown [23]. The occurrence of circulating immune complexes in 50% of patients with FMF, complement consumption, defective inhibition of complement activation and uncontrolled release of TNF during the attacks have been described. Therefore an immune related mechanism has been suggested to be involved in the pathogenesis of FMF [24]. Immune mechanisms also play roles in FMF-associated vasculitis [24]. It is interesting to note the presence of immunoglobulins, complement C3 and fibrinogen in the skin and kidney biopsies in some of our patients.

FMF may predispose to HSV, PAN, and other forms of vasculitis, either by amplifying the inflammatory response to a subclinical insult or by predisposing to immune-mediated diseases as a consequence of immune regulations associated with FMF [25]. Further investigations of the inflammatory consequences of FMF may also help to elucidate the pathogenesis of different types of vasculitis [20].

We reported an HSV patient with RPGN whose severe course may be affected by the presence of MEFV mutation. We suggest that colchicine provide additive effect on his therapy.

## Competing interests

The authors declare that they have no competing interests.

## Authors' contributions

BS collected data, written and participated in the design study, SM: conceived of the study, and participated in its design and coordination, PE: participated in the design study and ODK: participated in the design study, SS: carried out the pathological studies. All authors read and approved the final manuscript.

## Consent

Written informed consent was obtained from the patient for publication of this case report and accompanying images. A copy of the written consent is available for review by the Editor-in-Chief of this journal.
